# Immune checkpoint inhibitor-induced myocarditis in cancer patients: a case report and review of reported cases

**DOI:** 10.1186/s40959-021-00114-x

**Published:** 2021-08-09

**Authors:** Emma Matzen, Lars Erik Bartels, Brian Løgstrup, Stine Horskær, Christina Stilling, Frede Donskov

**Affiliations:** 1 Department of Oncology, Aarhus University Hospital, Palle Juul-Jensens, Boulevard 99, 8200 Aarhus N, Denmark; 2 Department of Rheumatology, Aarhus University Hospital, Aarhus, Denmark; 3 Department of Cardiology, Aarhus University Hospital, Aarhus, Denmark; 4 Department of Pathology, Aarhus University Hospital, Aarhus, Denmark

**Keywords:** Checkpoint immunotherapy, Myocarditis, Immunosuppressive therapy

## Abstract

**Background:**

Immune checkpoint inhibitor (ICI) induced myocarditis is a rare, severe, and often fatal adverse event. Evidence to guide appropriate immunosuppressive therapy is scarce. We present a case of ICI-induced myocarditis and a review of ICI-induced myocarditis cases to determine the most effective immunosuppressive therapeutic strategy for ICI-induced myocarditis.

**Methods:**

A systematic search of PubMed was carried out for treatment of ICI-induced myocarditis. Reference lists from identified articles were manually reviewed for additional cases.

**Results:**

A total of 87 cases with ICI-induced myocarditis were identified. The majority were melanoma (*n* = 39), lung cancer (*n* = 19), renal cell cancer (*n* = 10), and thymoma cancer patients (*n* = 4). In 38 (44%) cases, patients received high-dose steroid treatment only. A total of 49 (56%) cases were treated with immunosuppressive agents other than steroid; a total of 13 different immunosuppressive agents were used, including alemtuzumab or abatacept. The median time to onset of symptoms after initiation of ICI was 16 days (range, 1–196 days); cardiotoxic symptoms developed after 2 cycles of ICI (range, 1–13 cycles). A total of 48% of cases were fatal. In cases treated with high-dose steroids only vs. cases treated with other immunosuppressive agents, fatality was 55% and 43% respectively. In 64 out of the 87 cases, tumor control was not described. In patients treated with high-dose steroids only, two patients had stable disease as best tumor response; in patients treated with other immunosuppressive agents, one complete response, one partial response and seven stable disease were noted as best tumor response. Overall, 11 studies were at low risk of bias (12.6%), 38 at moderate risk of bias (43.7%) and 38 at high risk of bias (43.7%).

**Conclusion:**

Immune checkpoint inhibitor induced myocarditis is a serious and often fatal adverse event. High-dose prednisolone, alemtuzumab or abatacept are all possible treatments options for ICI-induced myocarditis, whereas infliximab increases the risk of death from cardiovascular causes, and should be avoided. Further research is needed.

## Background

Immune checkpoint inhibitor (ICI) therapy treatment has revolutionized the treatment of cancer. Checkpoint inhibitors are monoclonal antibodies targeting regulation receptors in the immune system such as programmed cell death receptor 1 (PD-1), programmed cell death ligand 1 (PD-L1), and cytotoxic T-lymphocyte-associated protein 4 (CTLA-4); binding to the inhibitory receptor mediates the immune system to attack and destroy cancer cells. ICI has shown activity in a large number of solid tumor and hematological malignancies, and is today the backbone therapy in many diseases [1].

ICI therapy may be associated with immune-related adverse events (irAE). A rare irAE is ICI-induced myocarditis, which is associated with a high mortality [2]. Mortality is most frequent in ICI combination therapy compared to ICI monotherapy [2].

There remains a lack of evidence to guide appropriate immunosuppressive therapy in ICI-induced myocarditis, as current guidelines are based on expert consensus; no randomized controlled studies have been performed to evaluate the efficiency of immunosuppressive therapeutic strategies. In the event of Common Toxicity Criteria for Adverse Events (CTCAE) of any grade, current guidelines recommend discontinuation ICI therapy and treatment with high-dose corticosteroids; if insufficient effect of steroid treatment, other immunosuppressive agents are suggested, such as antithymocyte globulin, mycophenolate mofetil, infliximab or tacrolimus [3]. Moreover, betablockers and angiotensin converting enzyme (ACE) inhibitors are indicated in patients with reduced LVEF according to heart failure guidelines [4]. However, the efficacy and safety of treatments for ICI-induced myocarditis have not been evaluated.

We present a case of ICI-induced myocarditis in a patient with metastatic renal cell carcinoma treated with checkpoint immunotherapy. Furthermore, we performed a systematic review of all known cases with ICI-induced myocarditis and reviewed the immunosuppressive treatments to identify the most effective strategy to attenuate toxicity while maintaining tumor control.

## Case presentation

**Fig. 1:**
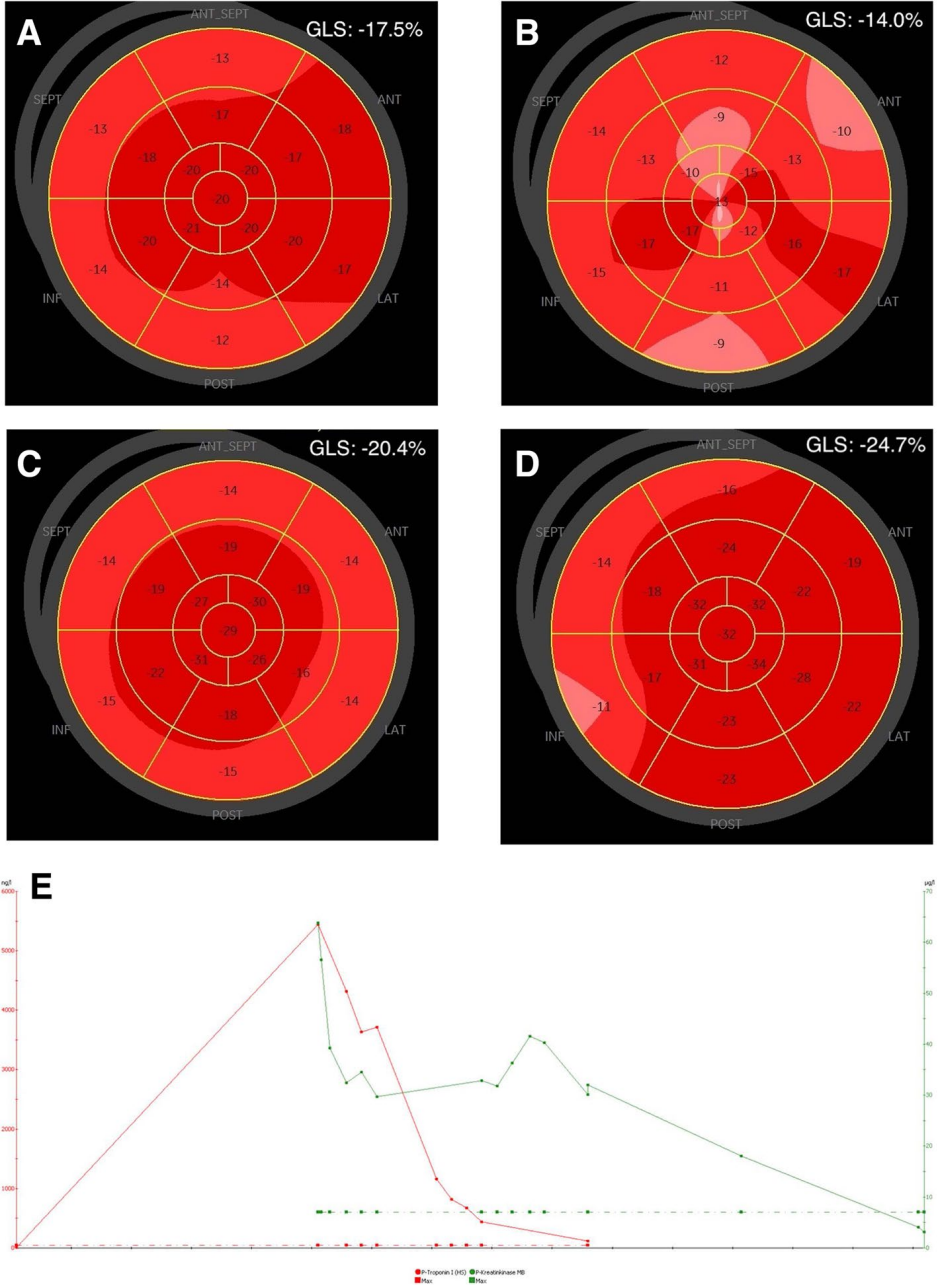
Two-dimensional transthoracic echocardiography (TTE) with global longitudinal strain (**A**-**D**) analysis and cardiac biomarkers (**E**). **A** TTE performed at the day of admission at the department of cardiology, at debut of symptoms, (cycle 2 day 12). **B** TTE performed on the day of peak of cardiac biomarkers, (cycle 2 day 14). **C** TTE performed approximately 1 year before admission. **D** TTE performed at the day of discharge, (cycle 2 day 21). **E** Cardiac biomarkers showing troponin peak. Troponin and CK-MB declined after initiation of high-dose steroid

**Fig. 2:**
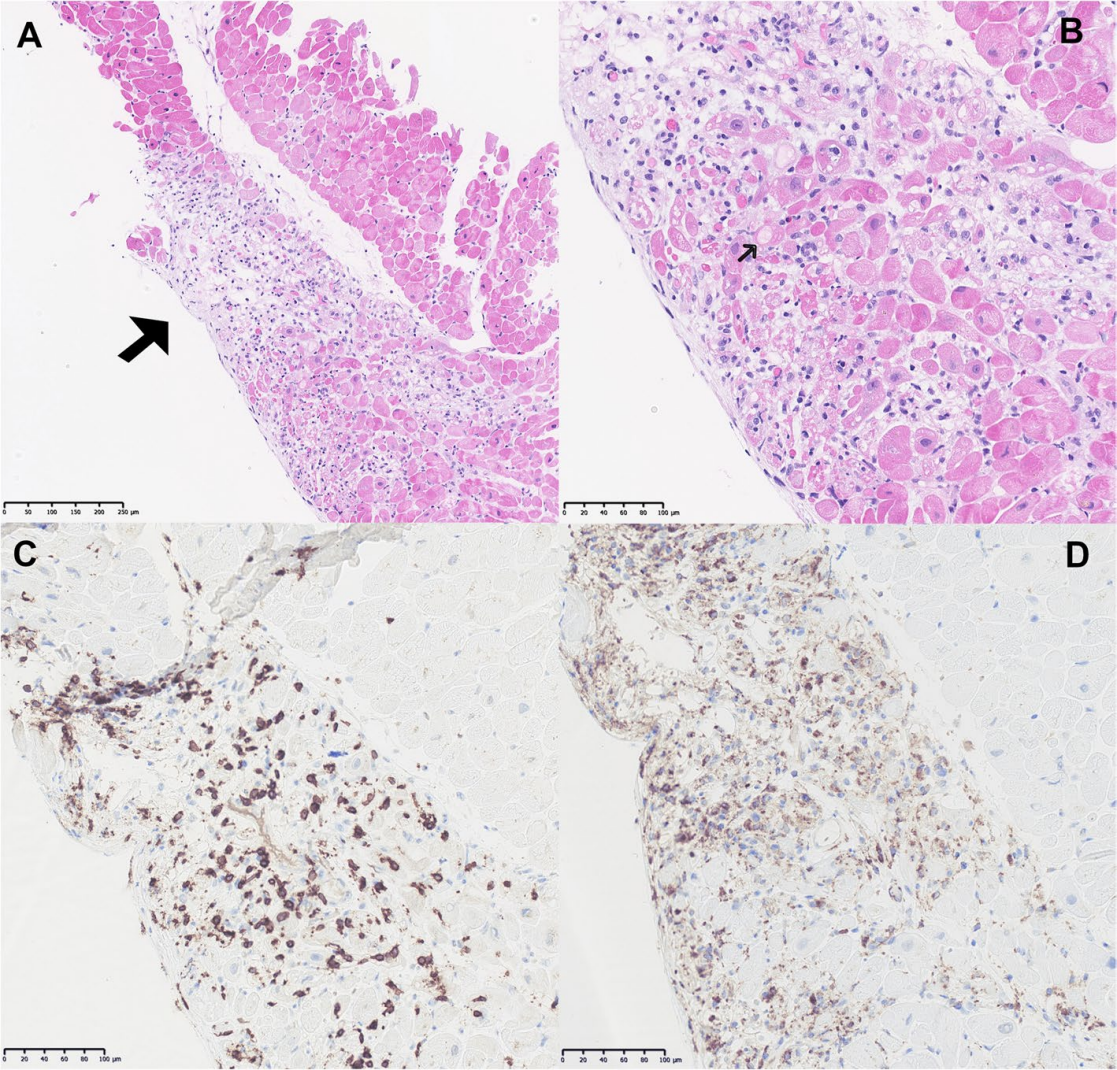
Pathological characteristics of immune checkpoint inhibitor-induced myocarditis. **A** Hematoxylin and eosin stain of patchy inflamed myocardium × 10 (arrow). **B** Hematoxylin and eosin stain of inflamed myocardium with necrotic myocytes × 20 (arrow). **C** CD3-positive T-lymphocytes, immunohistochemical staining × 20. **D** CD68-positive macrophages, immunohistochemical staining × 20

A 68-year-old man was diagnosed with synchronous metastatic renal cell carcinoma, with a primary tumor in the right kidney and metastases in the left adrenal gland, liver, lungs, pleurae and right chest wall. Blood tests showed anemia, elevated platelets, neutrophils, and corrected calcium; according to the International Metastatic Renal Cell Carcinoma Database Consortium (IMDC) [5] the patient was categorized in the poor risk prognostic group. The patient was treated with nivolumab (240 mg i.v.) and ipilimumab (88 mg i.v.). Twelve days after the second cycle of immunotherapy, the patient presented with dyspnea, peripheral edema, dizziness and chest pain, and was admitted to the emergency department. Biochemistry showed elevated cardiac biomarkers, TnI 5888 (ref. value < 24 ng/l) (Fig. 1) and CK-MB 56.6 (ref. value < 7 $\mu$g/l) and ALAT 309 (ref. value < 70 U/l), suggestive of myocarditis and hepatitis. Electrocardiogram (ECG) showed a 3-degree atrio-ventricular block. A transthoracic echocardiogram (TTE) (cycle 2 day 12) showed slightly affected global longitudinal strain (GLS) of left ventricular function at -17.5% (normal < -18.0%), left ventricular ejection fraction (LVEF) was 50% (normal > 60%), (Fig. 1A). Antibiotic treatment was started due to fever and elevated infection markers, CRP 43 (ref. value < 8 mg/l) and leucocytes 16 (ref. value 3.5–10.0 10^9/l). Immunotherapy was discontinued. The patient started treatment the following day (cycle 2 day 13) with prednisolone 100 mg daily. The patient was acutely transferred to a high facility Department of Cardiology at Aarhus University Hospital for a temporary pacemaker due to total atrio-ventricular block. A repeated TTE (cycle 2 day 14) showed clearly affected GLS of left ventricular function at -14.0% (Fig. 1B); for comparison a TTE performed a year before admission showed normal GLS -20.4% and EF 60% (Fig. 1C). Pulmonary embolism was excluded by an angio-CT of the thorax. Coronary angiography showed no significant findings. Due to the temporary pacemaker no cardiac MRI was performed. Endomyocardial biopsy obtained 3 days after presentation of symptoms (cycle 2 day 15) revealed severe myocyte necrosis with pronounced, patchy inflammation dominated by CD3-positive T-lymphocytes and occurrence of CD68-positive macrophages, consistent with the diagnosis of lymphocytic myocarditis (Fig. 2). Other causes of myocarditis (i.e., bacterial myocarditis, giant cell myocarditis, eosinophile myocarditis, CMV associated myocarditis and sarcoidosis) were excluded*.* Cardiac biomarkers kept declining on prednisolone treatment and sinus rhythm returned and treatment with the temporary pacemaker was terminated. A new TTE (cycle 2 day 21) showed improved cardiac function with a GLS of -24.7% (Fig. 1D). The patient was discharged in his normal condition.

Twelve days later he was readmitted due to dyspnea. ECG showed atrial flutter and metoprolol 50 mg daily and digoxin was initiated. Cardiac biomarkers were near normalized. The patient’s clinical condition decreased, and due to infection, he started antibiotics. CT revealed progression of metastases in the lung, mediastinal lymph nodes and liver. The patient’s condition continued to decline and he eventually expired, approximately 8 and a half weeks after the second dose of immunotherapy.

## Methods

### Information and search strategy

A systematic search in the medical database PubMed was conducted using the terms “checkpoint immunotherapy” and “checkpoint inhibitor immunotherapy-associated myocarditis” and “treatment”. The search included 1^st^ of January 2000 to the 22^nd^ of January 2021. Reference lists from identified articles were manually reviewed for additional cases.

### Eligibility criteria

A literature search involving studies of patients with ICI-induced myocarditis treated with immunosuppressive agents was conducted to answer the predefined PICO question:

Is it possible to improve the treatment of ICI-induced myocarditis by integrating an immunosuppressive agent as an alternative or supplement to prednisolone?

- Population – Cancer patients treated with checkpoint inhibitors (PD-1-, PD-L1- or CTLA-4-inhibitor) and adverse event with myocarditis.
- Intervention – Immunosuppressive therapy other than steroid, added to steroid-treatment – either mono or combination therapy.
- Comparators – steroid mono therapy vs. other immunosuppressive therapy.
- Outcome(s) — Primary outcome: death. Secondary outcome: sustained cardiac function. Third outcome: maintained tumor control.

### Study selection

The screening program Covidence was used to screen articles based on title and abstract to identify potential studies. Articles for full-text screening were retrieved online and evaluated for eligibility.

### Risk of bias (methodological quality) assessment of included studies

Risk of bias (methodological quality) was assessed using a tool developed by Murad et al. [6], to evaluate the methodological quality/risk of bias of case reports and case series based on the following domains: selection, ascertainment, causality and reporting. The domains contain a total of 8 questions; we removed the 5^th^ question concerning challenge/re-challenge phenomena, as the included cases did not describe any challenge/re-challenge phenomena, rendering 7 questions to address in this analysis. The questions were answered with a binary response to indicate whether the report was suggestive of bias or not. The quality of the report was considered good (low risk of bias), when all 7 criteria were fulfilled, moderate when 6 were fulfilled and poor when 5 or less were fulfilled (Table 1). First author assessed the risk of bias of the included studies.

**Table 1:** Risk of bias assessment of included studies

First author	Q 1		Q 2		Q 3		Q 4		Q 6		Q 7		Q 8		Risk of bias
	**Y**	**N**	**Y**	**N**	**Y**	**N**	**Y**	**N**	**Y**	**N**	**Y**	**N**	**Y**	**N**	
Doms [7]		**X**	**X**		**X**		**X**		**X**		**X**		**X**		Moderate
Esfahani [8]		**X**	**X**		**X**		**X**		**X**		**X**		**X**		Moderate
Salem [9]		**X**	**X**		**X**		**X**		**X**		**X**		**X**		Moderate
Matzen [10]	**X**		**X**		**X**		**X**		**X**		**X**		**X**		Low
Läubli [11]		**X**	**X**		**X**		**X**		**X**		**X**		**X**		Moderate
Zimmer [12]	**X**		**X**		**X**			**X**	**X**		**X**		**X**		Moderate
Behling [13]		**X**	**X**		**X**		**X**		**X**		**X**		**X**		Moderate
Semper [14]	**X**		**X**		**X**		**X**		**X**		**X**		**X**		Low
Mahmood [15]		**X**	**X**		**X**		**X**		**X**		**X**			**X**	High
Jain [16]		**X**	**X**		**X**			**X**	**X**		**X**		**X**		High
Liu [17]		**X**	**X**		**X**		**X**		**X**		**X**		**X**		Moderate
Frigeri [18]		**X**	**X**		**X**		**X**		**X**		**X**		**X**		Moderate
Norwood [19]		**X**	**X**		**X**		**X**		**X**		**X**		**X**		Moderate
Arangalage [20]		**X**	**X**		**X**		**X**	**X**	**X**		**X**			**X**	High
Yogasundaram [21]		**X**	**X**		**X**		**X**		**X**		**X**		**X**		Moderate
Tay [22]		**X**	**X**		**X**		**X**		**X**		**X**		**X**		Moderate
Tadokoro [23]		**X**	**X**		**X**		**X**		**X**		**X**		**X**		Moderate
Gibson [24]		**X**	**X**		**X**		**X**		**X**		**X**		**X**		Moderate
Guiney [25]		**X**	**X**		**X**			**X**	**X**		**X**		**X**		High
Osnat [26]		**X**	**X**		**X**		**X**		**X**		**X**		**X**		Moderate
Mehta [27]		**X**	**X**		**X**		**X**		**X**		**X**		**X**		Moderate
Reuben [28]		**X**	**X**		**X**		**X**		**X**		**X**			**X**	High
Tajmir-Riahi [29]		**X**	**X**		**X**		**X**		**X**		**X**		**X**		Moderate
Berg [30]		**X**	**X**		**X**		**X**		**X**		**X**		**X**		Moderate
Samara [31]		**X**	**X**		**X**			**X**	**X**		**X**		**X**		High
Khoury [32]		**X**	**X**		**X**			**X**	**X**		**X**		**X**		High
Katsume [33]		**X**	**X**		**X**		**X**		**X**		**X**		**X**		Moderate
Yanase [34]		**X**	**X**		**X**		**X**		**X**		**X**		**X**		Moderate
Reddy [35]		**X**	**X**		**X**		**X**		**X**		**X**			**X**	High
Ganatra [36]		**X**	**X**		**X**		**X**		**X**		**X**		**X**		Moderate
Fukusawa [37]		**X**	**X**		**X**		**X**		**X**		**X**		**X**		Moderate
Heinzerling [71] Case 1,5 & 8	**X**		**X**		**X**		**X**		**X**		**X**		**X**		Low
Imai [38]		**X**	**X**		**X**		**X**		**X**		**X**			**X**	High
Johnson [39] Case 1 & 2		**X**	**X**		**X**			**X**	**X**		**X**		**X**		High
Nasr [40]		**X**	**X**		**X**		**X**		**X**		**X**		**X**		Moderate
Rota [41] Case 1 & 2		**X**	**X**		**X**		**X**		**X**		**X**		**X**		Moderate
Yamaguchi [42]		**X**	**X**		**X**		**X**		**X**		**X**		**X**		Moderate
Chen [43]		**X**	**X**		**X**		**X**		**X**		**X**		**X**		Moderate
Nierstedt [44]		**X**	**X**		**X**		**X**		**X**		**X**			**X**	High
Christina [45]		**X**	**X**		**X**				**X**		**X**		**X**		High
Copeland-Halperin [46]		**X**	**X**		**X**		**X**		**X**		**X**			**X**	High
Gallegos [47]		**X**	**X**		**X**			**X**	**X**		**X**		**X**		High
Inayat [48]		**X**	**X**		**X**		**X**		**X**		**X**				Moderate
Lopez [49] Case 1		**X**	**X**		**X**		**X**		**X**		**X**			**X**	High
Lopez [49] Case 2		**X**	**X**		**X**			**X**	**X**		**X**			**X**	High
Martinez-Calle [50]		**X**	**X**		**X**		**X**		**X**		**X**		**X**		Moderate
Monge [51]		**X**	**X**		**X**			**X**	**X**		**X**		**X**		High
Sakai [52]		**X**	**X**		**X**		**X**		**X**		**X**			**X**	High
Balanescru [53] Case 1	**X**		**X**		**X**		**X**		**X**		**X**		**X**		Low
Balanescru [53] Case 2 & 3	**X**		**X**		**X**		**X**		**X**		**X**			**X**	Moderate
Guo [54] Case 1 & 3	**X**		**X**		**X**		**X**		**X**		**X**		**X**		Low
Guo [54] Case 2 & 5	**X**		**X**		**X**			**X**	**X**		**X**		**X**		Moderate
Bukamur [55]		**X**	**X**		**X**			**X**	**X**		**X**			**X**	High
Agrawal [56] Case 1,2 & 3	**X**		**X**		**X**		**X**		**X**		**X**		**X**		Low
Agrawal [56] Case 4	**X**		**X**		**X**			**X**	**X**		**X**			**X**	High
Agrawal [56] Case 5	**X**		**X**		**X**		**X**		**X**		**X**			**X**	Moderate
Arora [57] Case 1,2,3,7		**X**	**X**		**X**			**X**	**X**		**X**			**X**	High
Arora [57] Case 4		**X**	**X**		**X**		**X**		**X**		**X**			**X**	High
Arora [57] Case 8		**X**	**X**		**X**			**X**	**X**		**X**		**X**		High
Xing [58]		**X**	**X**		**X**			**X**	**X**		**X**		**X**		High
Kimura [59]		**X**	**X**		**X**		**X**		**X**		**X**		**X**		Moderate
Fazel [60]		**X**	**X**		**X**			**X**	**X**		**X**		**X**		High
Hardy [61]		**X**	**X**		**X**		**X**		**X**		**X**		**X**		Moderate
Saibil [62]		**X**	**X**		**X**		**X**		**X**		**X**			**X**	High
Ansari-Gilani [63]Case 1& 2		**X**	**X**		**X**		**X**		**X**		**X**		**X**		Moderate
Ansari-Gilani [63] Case 3		**X**	**X**		**X**			**X**	**X**		**X**		**X**		High
McDowall [64]		**X**	**X**		**X**		**X**		**X**		**X**		**X**		Moderate
Shah [65]	**X**		**X**		**X**			**X**	**X**		**X**			**X**	High
Joseph [66]	**X**		**X**		**X**			**X**	**X**		**X**			**X**	High
Zhang [67]	**X**		**X**		**X**			**X**	**X**		**X**			**X**	High
Konstantina [68] Case 1		**X**	**X**		**X**		**X**		**X**		**X**			**X**	High
Konstantina [68] Case 2		**X**	**X**		**X**		**X**		**X**		**X**			**X**	High
Wang [69]		**X**	**X**		**X**			**X**	**X**		**X**		**X**		High
von Itzstein [70]		**X**	**X**		**X**			**X**	**X**		**X**		**X**		High
Total: 87 cases	**20**	**67**	**67**		**67**		**59**	**29**	**87**		**87**		**62**	**25**	Low risk: 11 Moderate risk: 38 Moderate risk: 38

*Y:* yes, *N:* no, *Q:* question

Question 1: Does the patient(s) represent(s) the whole experience of the investigator (centre) or is the selection method unclear to the extent that other patients with similar presentation may not have been reported?

Question 2: Was the exposure adequately ascertained?

Question 3: Was the outcome adequately ascertained?

Question 4: Were other alternative causes that may explain the observation ruled out?

Question 5: Was there a challenge/re-challenge phenomenon?

Question 6: Was there a dose–response effect?

Question 7: Was follow-up long enough for outcomes to occur?

Question 8: Is the case(s) described with sufficient details to allow other investigators to replicate the research or to allow practitioners make inferences related to their own practice?

## Results

### Study characteristics

**Fig. 3:**
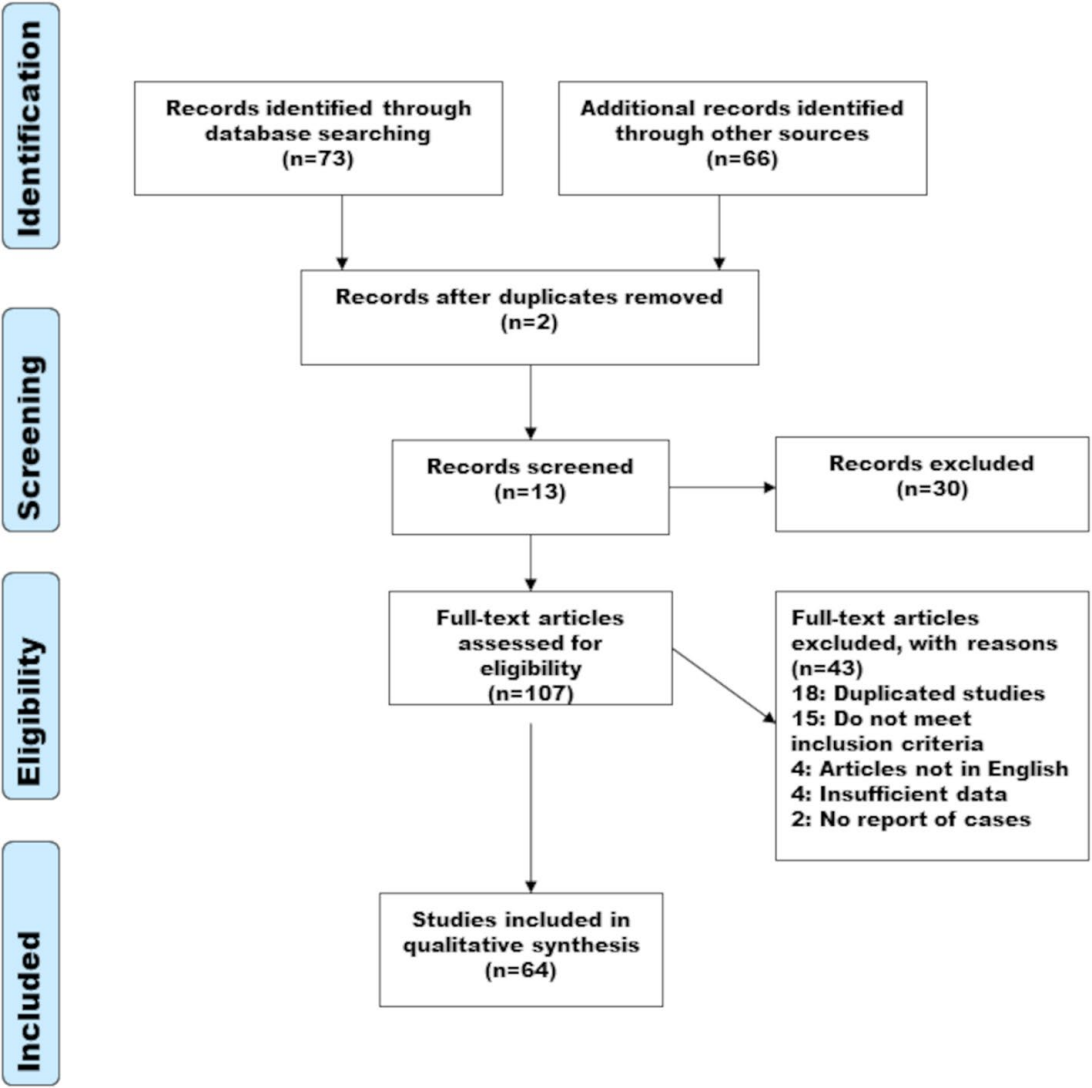
PRISMA flow diagram: The selection process including exclusion criteria

The flow diagram of the study selection is shown in Fig. 3. The literature search resulted in the retrieving of 73 publications. Sixty-six articles were added during manual search of the bibliographies of identified articles. Of the 139 articles, 2 were duplicates, leaving 137 articles for screening. One hundred seven publications were potentially eligible after title- and abstract screening. Full text screening was made on 107 articles and 43 articles were excluded for the following reasons: Fifteen publications did not meet inclusion criteria, 4 articles were not written in English, 2 articles did not contain any report of cases, 4 articles contained insufficient data in the case reports and finally, 18 studies were duplicated. A total 66 articles containing case reports were included in the qualitative synthesis. The publication date of the included studies ranged from 2015 to 2021.

### Risk of bias assessment

The results of risk of bias assessment of included studies are shown in Table 1.

Eleven studies were at low risk of bias (12.6%), 38 at moderate risk of bias (43.7%) and 38 at high risk of bias (43.7%). For question 1 (Table 1), 67 studies contained an unclear selection approach, as they did not mention whether the reported patient(s) represented the whole experience of the medical center and therefore had reported all known cases in their center. There could be a risk that patients with a similar presentation may not have been reported, as one could expect that mild asymptomatic cases with only biochemical evidence suggestive of myocarditis could be overlooked. The same could be mentioned in regards to serious cases of ICI-induced myocarditis, which may not have been reported for different reasons. Both could lead to selection bias.

For question 4, 29 studies did not describe whether alternative causes (e.g., viruses, other drugs or acute myocardial infarction etc.) that could give rise to similar symptoms were ruled out. For the domain of reporting (question 8) 25 studies did not describe the intervention with immunosuppressive agents with sufficient details (precise dosage of one or more of the immunosuppressive agents were not mentioned), which makes it difficult for readers to apply the evidence derived from the report in their practice.

### Patient characteristics

A total of 87 patient cases were included. All patients had a cancer diagnosis and were treated with ICIs (anti-PD-1, anti-PD-L1 and anti-CTLA-4-inhibitor) mono or combination therapy. Thirty-nine patients were diagnosed with melanoma, 19 with lung cancer, 10 with renal cell carcinoma, 2 with prostate cancer, 4 with thymic carcinoma, 2 with urothelial carcinoma, 1 with endometrial cancer, 1 with glioblastoma, 1 with chronic myelomonocytic leukemia, 1 with metastatic sarcoma, 1 with metastatic breast cancer, 1 with metastatic gastric carcinoma, 1 with esophageal adenocarcinoma, 1 with myeloma and 1 with mesothelioma. Two patients were diagnosed with myelodysplastic syndrome (MDS), which is characterized as a pre-leukemic disease [53]. Other adverse events than myocarditis induced by ICI therapy reported in the studies were hepatitis (15 patients), myositis (24 patients), colitis (2 patients), thyroiditis (11 patients), myasthenia gravis (13 patients), pneumonitis (2 patients), rhabdomyolysis (2 patients), hypophysitis (3 patients), neuritis (1 patient), polyneuropathy (1 patient), conjunctivitis/uveitis (1 patient), Syndrome of inappropriate antidiuretic hormone (1 patient), encephalitis (1 patient) and Stevens-Johnson syndrome (1 patient). Adverse events due to the immunosuppressive regimens were not reported in any of the included studies.

The diagnosis of myocarditis was based on cardiac biomarkers, ECG, echocardiography, cardiac magnetic resonance or computer tomography of the heart, cardiac catherization and endomyocardial biopsy. Twenty studies included biopsy-verified myocarditis with findings of immune infiltration of the myocardium with CD4-positive T-lymphocytes, CD8-positive T-lymphocytes and CD68-positive macrophages [11, 16, 19, 22, 23, 26, 36–39, 42, 43, 47, 50, 61, 62, 67, 71]. One study included several biopsies performed during and after immunosuppressive therapy showing improvement with evidence of early repair, less inflammatory cells and patchy foci of fibrosis [22].

### Immunosuppressive treatment

Reported immunosuppressive regimens in the included studies consisted of high-dose glucocorticoids, antithymocyte globulin (ATG), mycophenolate mofetil, alemtuzumab, infliximab, abatacept, plasmapheresis, tocilizumab, immunoglobulin, rituximab, tacrolimus, methotrexate and cyclophosphamide. In 38 (44%) cases, the patients received high-dose steroid treatment only. The immunosuppressive regimens of the 49 (56%) cases consisted of treatment with one or more immunosuppressive agents other than steroids (Table 2). Effects of the immunosuppressive regimens were measured on improvement in clinical condition, cardiac biomarkers and LVEF.

**Table 2:** Immunosuppressive agents used in the included studies and cases

Intervention	Mechanism of action	Advantages	Disadvantages	Articles/cases
Steroid	Multifactorial immunosuppressive	Rapid immunosuppressive effectKnown to be potent suppressor of T cell function and activity	High-dose corticosteroids increase the risk of infections, gastric ulcer, exacerbate congestive heart failure, trigger/dys-regulate diabetes	[10–14, 23–25, 27–33, 36, 37, 39, 44–46, 48, 49, 51, 52, 54–57, 59, 63, ﻿^71^] (total 38)
Antithymocyte globulin	Polyclonal antibody induces T-lymphocyte depletion via complement-dependent cell lysis	Can induce large reductions (through cell lysis) in the number of circulating T-lymphocytes	After administration the patient becomes immune-deficient for a longer period of time. Risk of anaphylactic reactions.	[16, 22, 26, 49, 56, 57, 64, 67] (total: 11)
Mycophenolate mofetil	Inhibits the proliferation of T- and B-lymphocytes	Has a cytostatic effect on B- and T-lymphocytes	Risk of infections. Risk of reactivating hepatitis B or C, Epstein-Barr virus and Cytomegalovirus	[8, 15, 17, 21, 22, 26, 35, 54, 57, 64, 68] (total: 14)
Infliximab	Monoclonal antibody inhibits tumor necrosis factor (TNF-alfa)	Inhibits cell signaling that induces inflammation	Risk of serious infections	[18, 39, 47, 50, 56, 57, 62, 65] (total: 8)
Alemtuzumab	Monoclonal antibody that binds to CD52 on the surface of mature T- and B-lymphocytes, which induces their destruction through activation of the complement system	Induces rapid T-cell depletion	Risk of inducing autoimmune diseases and infections	[8] (total: 1)
Abatacept	A fusion protein that binds CD28/CD86, which modulates the co-stimulation of T-cells so they cannot be activated	Blocks the activation of T-cells	Risk of infections, risk of inducing malignancy	[9, 17] (total: 2)
Plasmapheresis	An extracorporeal therapy, which can remove immune complexes from the blood	Rapid removal of circulating immune complexes from the blood of the patient	Risk of infections	[8, 9, 17, 18, 20, 21, 34, 40, 58, 61, 65, 66] (total: 12)
Tocilizumab	Humanized monoclonal antibody directed at the proinflammatory cytokine interleukin (IL)-6-receptor	Anti-inflammatory effect	Risk of gastrointestinal perforationRisk of infections	[7, 69] (total: 2)
Immuno-globulin	Induces inactivation of autoreactive T-cells, inactivation of B-cells, regulation and antibody production. Interferes with complement activation	Has several immunosuppressive effects	Risk of acute kidney injury and thromboembolic events	[18, 34, 38, 40–43, 53, 56, 58, 60, 65, 66, 68–70] (total: 18)
Rituximab	Monoclonal antibody directed towards CD20 on B-cells, binding causes death of the cell and B-cell depletion	Induces depletion of B-cells	Increased infections and rare opportunistic infections, such as PML, have been reported	[8, 68] (total: 2)
Tacrolimus	Inhibits calcineurin, which is involved in the production of IL-2. IL-2 promotes the development and proliferation of T-cells	Inhibits development and proliferation of T-cells	Risk of infections, kidney and liver toxicity and blindness	[20, 38] (total: 2)
Methotrexate	Antimetabolite that inhibits the enzyme dihydrofolate, which leads to a lack of tetrahydrofolate and thereby inhibits the DNA-synthesis of cells	Suppressing of the functions of T-cells	Risk of infections, pulmonary side effects and hepatotoxity	[40] (total: 1)
Cyclophos-phamide	Cyclophosphamide is an alkylating agent with cytostatic effect. It binds the RNA and DNA of the cell, leading to the inactivation of transcription and translation	Affects both the humoral and the cellular immune response	Bone marrow suppression	[49, 57] (total: 2)

### Outcomes

The median time to onset of symptoms after initiation of ICI was 16 days, but with a wide variation of 1 to 196 days. Number of ICI-cycles was described in 63 of the cases. In average cardiotoxic symptoms developed after 2 cycles of ICI with a variation of 1 to 13 cycles. Fatal outcome was observed in 48% of the cases. Death was caused by cardiac complications, infections or clinical deterioration. Follow-up in the cases had a wide variation as some cases did not have any follow-up after the patients were discharged and others included a follow-up after several months.

Twenty-one of the 38 patients (55%), treated with high-dose steroids only, died. Five patients died of sudden cardiac arrest, 3 patients expired due to arrhythmia, 1 patient died due to infection and 12 patients experienced clinical deterioration and were transitioned to comfort care. The remaining 17 patients out of the 38 (45%) showed improvement in clinical condition. Seven patients had improved LVEF and 3 patients had improved biochemical markers (declining cardiac biomarkers). Of the 49 cases treated with one or more immunosuppressive agents other than steroid, 21 (43%) were fatal. Twenty-eight out of the 49 patients (57%) showed improved clinical condition with immunosuppressive treatment. Six patients had improvement in cardiac biomarkers with declining/normalized troponin levels, 4 patients had improvement in ECG and 8 patients showed improved LVEF. Comparing fatality in the cases, 55% treated with high-dose steroids only were fatal vs. 43% fatality in cases treated with other immunosuppressive agents.

The immunosuppressive therapy regimens in the cases treated with immunosuppressive agents other than steroid (23 out of 49) varied, as only 3 cases were not treated with steroid initially, but with only one immunosuppressive agent being either ATG (1 patient) [67] or immunoglobulin (3 patients) [53]. The other 20 cases were treated with steroid initially, followed by one to four other immunosuppressive agents due to lack of improvement in clinical condition, cardiac biomarkers, ECG or LVEF [7–9, 15, 17–20, 22, 26, 34, 35, 41, 42, 54, 56, 58, 69, 70].

In 64 out of the 87 cases, tumor control was not described. In the patients treated with high-dose steroids only, two patients had stable disease [23, 59] and seven patients showed progression of metastatic disease [10, 13, 54, 63, 71]. In the cases treated with one or more immunosuppressive agents other than steroid, complete tumor response was reported in one patient [8] and partial tumor response was reported in one patient [34]. Stable disease was reported in seven patients [8, 9, 17, 19, 22, 23, 56]. Progression of disease was reported in seven patients [15, 38, 53, 64, 65, 70].

## Discussion

Checkpoint immunotherapy has revolutionized the treatment options in oncology. ICI-induced myocarditis is a rare, but serious, and often fatal adverse event. To our knowledge, this is the largest review of 87 cases of ICI-induced myocarditis treated by corticosteroids alone or immunosuppressive therapy other than corticosteroids, where various immunosuppressive therapy regimens were used. It is evident that better treatment guidelines are needed when it comes to treatment of ICI-induced myocarditis.

The median time to onset of symptoms after initiation of ICI was 16 days (range, 1–196 days) equivalent to two cycles of ICI (range, 1–13 cycles). Mahmood et al. reported a median time of onset of myocarditis 34 days after first ICI dose [72]. Escudier et al. reported a median time of 65 days from initiation of ICI to presentation of cardiotoxic effects, but also with a wide variation of 2 to 454 days [73]. Hence ICI-myocarditis should still be considered even though the patients have received several cycles of ICI therapy. We observed 48% with fatal outcome. Other studies have reported a fatality rate of 50% of myocarditis cases, with fatality being most frequent in ICI combination therapy (65.5%) [2]. Follow-up in the cases had a wide variation as some cases did not have any follow-up after the patients were discharged and others included a follow-up of several months. This makes it difficult to make an assessment of the long-term effect of the immunosuppressive regimens in some of the cases in regards to the cardiac function.

Myocarditis is an inflammatory disease of the myocardium caused by a broad variety of infectious and autoimmune conditions. Myocarditis is microscopically categorized according to the predominant histopathologic pattern generally reflecting different aetiologies and pathogenic mechanisms, which could impact the choice of treatment. In the fulminant phase of lymphocytic myocarditis, myocyte damage/necrosis is a common histopathologic finding, which is gradually substituted by fibrosis in the healing process [74]. One possible pathophysiologic mechanism of ICI-myocarditis is that cardiac myocytes may share targeted antigens with the tumor, therefore becoming targets of activated T-lymphocytes resulting in lymphocytic infiltration of the myocardium [39]. Myocarditis can cause several cardiovascular complications such as arrhythmias, myocardial infarction, heart failure or cardiogenic shock. Outcome is favorable in half of the patients, but 25% develop persistent cardiac dysfunction. Myocarditis can also result in sudden cardiac death, fulminant heart failure or progressive dilated cardiomyopathy [75]. Hence immediate initiation of immunosuppressive treatment is vital to avoid irreversible immune-mediated damage to the heart. An important biopsy finding in our study was that the pathological finding of ICI-induced myocarditis is identical to lymphocytic myocarditis in general. Twenty of the reported cases contained endomyocardial biopsies, showing lymphocytic infiltration within the myocardium primarily comprising T-lymphocytes; consistent with our findings and with the diagnosis lymphocytic myocarditis. A study by Escudier et al. reported lymphocytic myocarditis on biopsies in 8 out of 9 patients [73]. Immunosuppressive agents that target T-lymphocytes could therefore be a suitable therapy for ICI-induced myocarditis based on the histopathologic pattern of lymphocytic myocarditis and from an immunologic point of view. Hence, high-dose corticosteroids may be first line therapy for ICI-induced myocarditis and should be initiated promptly, and if there is no rapid effect of steroid treatment after a few days, other immunosuppressive agents should be promptly added in order to minimize immune-mediated destruction of the myocardium and subsequent damage to the heart. We observed a lower fatality in cases treated with immunosuppressive agents other than steroid compared to cases treated with steroid only (43 vs. 55%), which suggests that outcome improves by adding other immunosuppressive agents, when steroid treatment alone has insufficient clinical effect. However, the overall observed fatality of the cases was high (48%), which could indicate that immunosuppressive treatment in general was not initiated rapidly enough; a better outcome would probably have been reported if immunosuppressive treatment were initiated faster. Future research may clarify whether non-steroids may be initiated initially, omitting steroids.

Esfahani et al. reported the first case of treatment with alemtuzumab, a CD52-inhibitor. Treatment led to rapid cytolytic immunosuppression with resolution of cardiac immune toxic effects and sustained complete tumor response at 4-month follow-up [8]. Thus, using alemtuzumab as a second line treatment may be an option due to its ability to rapidly deplete T-lymphocytes from the circulation and hence minimize autoimmune damage to the myocardium. However, so far treatment with alemtuzumab has only been reported in one case. Further research is warranted.

Two cases of treatment with abatacept were reported by Salem et al. and Liu et al. [9, 17]. In the case by Salem et al. treatment with abatacept led to rapid decrease in troponin levels and symptoms with persisting tumor control after one month of administration of abatacept [9]. In the case by Liu et al. abatacept treatment improved the patient’s functional status, however troponin levels were persistently elevated despite 6.5 months of immunosuppressive treatment. Surveillance CT showed tumor control [17]. These findings could suggest that treatment with alemtuzumab or abatacept as second line therapy could be effective in treating ICI-induced myocarditis as both substances target T-lymphocytes, which are the predominant finding in endomyocardial biopsies from patients with ICI-induced myocarditis, as reported in both this and previous studies [11, 16, 19, 22, 23, 26, 36–39, 42, 43, 47, 50, 61, 62, 67, 71]. Furthermore treating ICI-induced myocarditis with alemtuzumab or abatacept could have a positive outcome in regard to tumor control compared to other immunosuppressive agents. On the contrary, treating ICI-induced myocarditis with infliximab (monoclonal antibody that inhibits tumor necrosis factor alpha) increases the risk of death from cardiovascular causes with odds ratio 12.0, as was reported in a recent study by Cautela et al. [76]. We also observed that in cases treated with infliximab 3 out of 8 patients (37.5%) died of cardiovascular causes [39, 47, 50]. Thus, protecting the heart, but not losing the value of the immunotherapy on the cancer, is the optimal balance; that was demonstrated sadly in our patient, that only received high-dose corticosteroid treatment.

Treatment with alemtuzumab and abatacept can both give rise to infections and there is a risk of inducing autoimmune diseases when treating with alemtuzumab. However, these adverse events do not especially differ from adverse events induced by other immunosuppressive agents. Another advantage of immunosuppressive treatment with alemtuzumab or abatacept is that it would be possible to reduce steroid-doses, as prolonged high-dose steroid treatment can be toxic. Prospective studies evaluating the most efficient treatment are warranted.

**Fig. 4:**
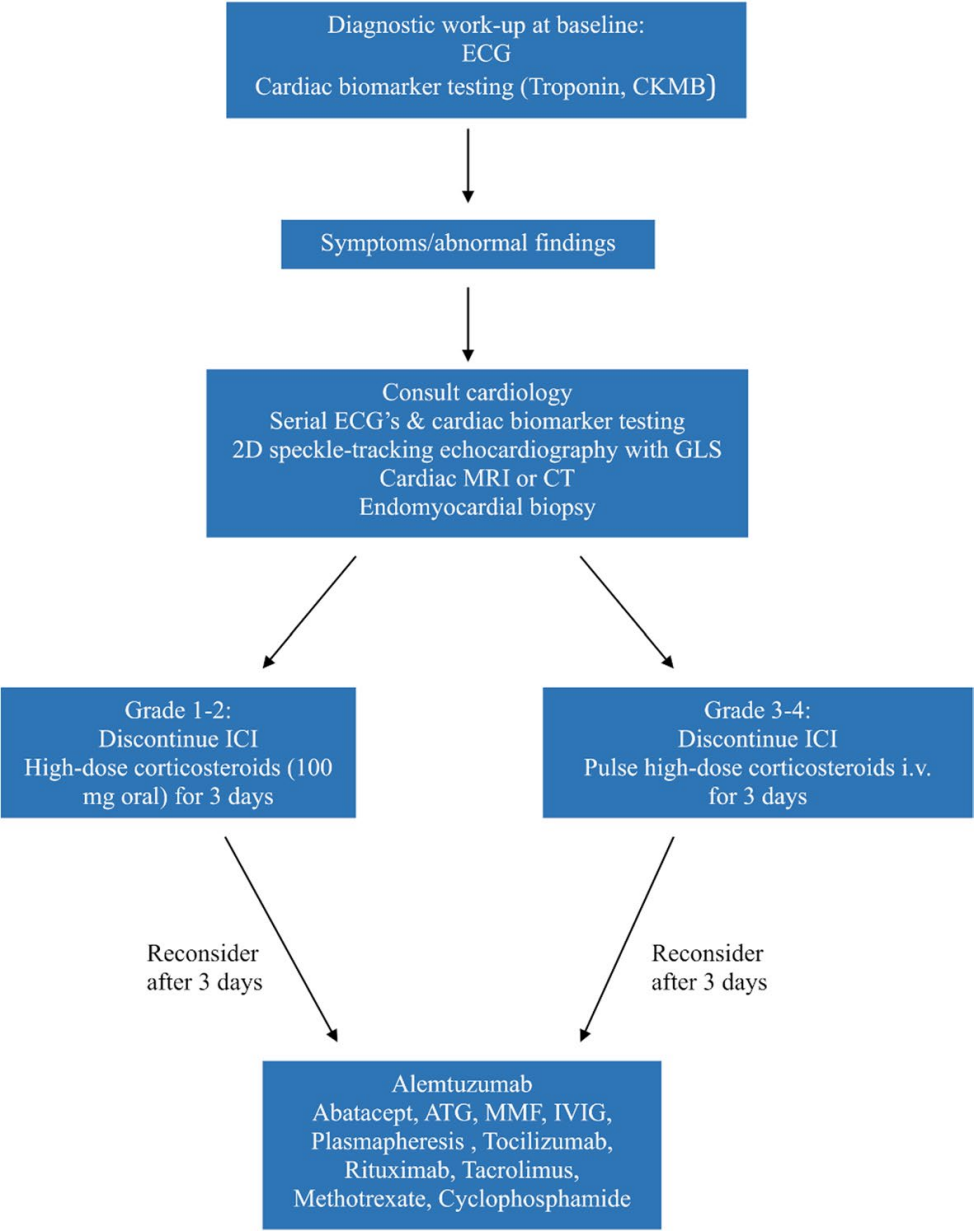
Assessment and management guideline for ICI induced myocarditis

All myocarditis CTCAE grades warrant work-up and intervention given potential for cardiac compromise. The current ASCO guideline [3] recommend that patients treated with ICIs are monitored with biochemical markers (cardiac biomarkers) and ECG at baseline. If these are abnormal or the patients become symptomatic, cardiology consultation should be pursued with serial ECGs and cardiac biomarker testing. 2D speckle-tracking echocardiography with GLS should be performed, as some patients with myocarditis show preserved LVEF. Other diagnostics should include cardiac MRI or CT and endomyocardial biopsy [77]. The endomyocardial biopsy is the gold-standard test for the diagnosis of myocarditis [78, 79]. The clinical mindset should be an aggressive approach to diagnosis with MRI and/or endomyocardial biopsy, and if myocarditis is found, treatment could be initiated immediately, whether or not symptoms are present. Management of CTCAE grade 1 myocarditis (i.e., elevated cardiac biomarkers without symptoms) and CTCAE grade 2 myocarditis (i.e., symptoms with moderate activity), or persistent elevated cardiac biomarkers should include discontinuation of ICI treatment and treatment with oral high-dose corticosteroids (100 mg daily) for 3 days; if there is insufficient effect of steroid treatment clinicians should revaluate and consider adding other immunosuppressive agents. For the management of CTCAE grade 3 or 4 myocarditis pulse high-dose corticosteroids IV should be given for 3 days as a first line treatment; if there is clinical improvement consider reducing steroid doses (by 20 mg daily) and follow cardiac markers closely. If there is no clinical improvement after 3 days of high-dose corticosteroid treatment, other immunosuppressive agents should be added. Clinicians could use findings in endomyocardial biopsies to guide supplement immunosuppressive therapy (Fig. 4).

To our knowledge, based on clinicaltrials.gov, there are no on-going clinical trials evaluating treatments for ICI-induced myocarditis. However, the ASCO guideline is based on limited data and will be expected to change as more data are accrued.

This review has limitations mainly due to the reporting of case series only; this has generally the lowest grade of evidence. Moreover, the included information in the case reports varied considerably where only 13% had low risk of bias, according to the criteria by Murad et al. [6]. None of the included studies reported adverse events to the immunosuppressive therapies, which leaves the reader to wonder if it is possible that no adverse events were reported due to the large amounts of immunosuppressive agents used.

## Conclusion

Immune checkpoint inhibitor induced myocarditis is a serious and often fatal adverse event. High-dose prednisolone, alemtuzumab or abatacept are all possible treatments for ICI-induced myocarditis, whereas infliximab increases the risk of death from cardiovascular causes, and should be avoided. Further research is needed to determine the most effective immunosuppressive therapeutic strategy for ICI-induced myocarditis.

## Data Availability

The datasets used and/or analyzed during the current study are available from the corresponding author on reasonable request.
